# Supplementary material for: Quantum reference frames for an indefinite metric
Source: Commun Phys. 2023 Aug 26;6(1):231. doi: 10.1038/s42005-023-01344-4 (PMC11041732; doi:10.1038/s42005-023-01344-4)
Supplement: Supplementary file 1 — Supplementary Information [file 42005_2023_1344_MOESM1_ESM.pdf]

Supplementary Information:
Quantum reference frames for treating an indefinite spacetime metric

Anne-Catherine de la Hamette,[1,2,*] Viktoria Kabel,[1,2,*] Esteban Castro-Ruiz,[3,4] and Časlav Brukner[1,2]

[1] *Vienna Center for Quantum Science and Technology (VCQ), Faculty of Physics, University of Vienna, Boltzmanngasse 5, A-1090 Vienna, Austria*
[2] *Institute for Quantum Optics and Quantum Information (IQOQI), Austrian Academy of Sciences, Boltzmanngasse 3, A-1090 Vienna, Austria*
[3] *Institute for Theoretical Physics, ETH Zurich, Zurich, Switzerland*
[4] *Université Paris-Saclay, Inria, CNRS, LMF, 91190 Gif-sur-Yvette, France*

SUPPLEMENTARY NOTE 1: THE QRF FORMALISM

To provide the necessary background regarding the quantum reference frame (QRF) formalism, we summarize the most essential notions for the present work in this supplementary note. The concept of QRFs emerges from the observation that reference frames are essential and very often implicit in the description of physical phenomena. In classical mechanics and standard quantum theory, they are typically excluded from the description and treated as classical non-dynamical systems. In this approach, we take seriously the fact that reference frames are instantiated by the rods and clocks of our theory and should thus be explicitly included as physical and, in particular, quantum systems. As a consequence, states of quantum systems are generally defined relative to a QRF. By construction, the reference system is in the trivial state with respect to itself. In the case of the translation group, this corresponds to the position eigenstate $|x=0\rangle$. More generally, the trivial state $|e\rangle$, where e is the unit element of the symmetry group, is associated to the reference system [1].

Given the state of systems relative to one QRF, it is possible to change to the description with respect to another QRF. In order to preserve the probabilities of measurement outcomes, we require such a transformation to be unitary. We illustrate the QRF change operator in the simple case of three quantum systems A, B, and C characterized by their position. Let us therefore consider a Hilbert space $\mathcal{H}^{(\mathrm{A})}_{\mathrm{ABC}} = \mathcal{H}^{(\mathrm{A})}_{\mathrm{A}} \otimes \mathcal{H}^{(\mathrm{A})}_{\mathrm{B}} \otimes \mathcal{H}^{(\mathrm{A})}_{\mathrm{C}}$ of the three systems with respect to the reference frame of A. The QRF change operator $\hat{\mathcal{S}}^{\mathrm{A}\to\mathrm{B}}$ maps states in $\mathcal{H}^{(\mathrm{A})}_{\mathrm{ABC}}$ to states in $\mathcal{H}^{(\mathrm{B})}_{\mathrm{ABC}} = \mathcal{H}^{(\mathrm{B})}_{\mathrm{A}} \otimes \mathcal{H}^{(\mathrm{B})}_{\mathrm{B}} \otimes \mathcal{H}^{(\mathrm{B})}_{\mathrm{C}}$, that is, states relative to B. It is a quantum-controlled coordinate transformation in the sense that it conditions on the state of the original reference frame A in each branch and performs a state-dependent coordinate transformation on the rest of the systems, B and C. Finally, it exchanges the labels of A and B to ensure that the trivial state is associated to the new reference frame B. In the case of translations, the QRF change operator takes the following form [1]:

$$\hat{\mathcal{S}}^{\mathrm{A}\to\mathrm{B}} = \mathrm{SWAP}_{\mathrm{AB}} \circ \mathbb{1}_{\mathrm{A}} \otimes \int dx_i dx_j \, |{-x_i}\rangle\langle x_i|_{\mathrm{B}} \otimes |x_j - x_i\rangle\langle x_j|_{\mathrm{C}} \,. \tag{1}$$

This operator controls on the position of the new reference system B and shifts any third system C by the respective distance. It further reflects B about the origin and swaps the labels of A and B such that B ends in the trivial state. This transformation is equivalent to

$$\hat{\mathcal{S}}^{\mathrm{A}\to\mathrm{B}} = \hat{\mathcal{P}}_{\mathrm{AB}} \circ \mathbb{1}_{\mathrm{A}} \otimes e^{-\frac{i}{\hbar}\hat{x}_{\mathrm{B}}\hat{p}_{\mathrm{C}}} \tag{2}$$

as first introduced in [2]. The form of the exponential operator clearly shows that we perform a shift of system C by an amount controlled on the position of system B while $\hat{\mathcal{P}}_{\mathrm{AB}} \equiv \mathrm{SWAP}_{\mathrm{AB}} \circ \int dx \, |{-x}\rangle\langle x|_{\mathrm{B}}$ adjusts the states of systems A and B. Letting this QRF operator act on a general state

$$|\psi\rangle^{(\mathrm{A})}_{\mathrm{ABC}} = |0\rangle_{\mathrm{A}} \otimes \int dx_i dx_j \psi(x_i, x_j) \, |x_i\rangle_{\mathrm{B}} \, |x_j\rangle_{\mathrm{C}} \,, \tag{3}$$

it is straightforward to see that this yields

$$|\psi\rangle^{(\mathrm{B})}_{\mathrm{ABC}} = |0\rangle_{\mathrm{B}} \otimes \int dx_i dx_j \psi(x_i, x_j) \, |{-x_i}\rangle_{\mathrm{A}} \, |x_j - x_i\rangle_{\mathrm{C}} \,. \tag{4}$$

---

* These authors contributed equally.

Similarly, the QRF change operator allows to transform observables as

$$\hat{\mathcal{O}}^{(\mathrm{A})} \mapsto \hat{\mathcal{O}}^{(\mathrm{B})} = \hat{\mathcal{S}}^{\mathrm{A}\to\mathrm{B}} \hat{\mathcal{O}}^{(\mathrm{A})} \left(\hat{\mathcal{S}}^{\mathrm{A}\to\mathrm{B}}\right)^{\dagger}. \tag{5}$$

Note that this way, the observable expectation values remain the same in every frame, that is

$${}^{(\mathrm{A})}_{\mathrm{ABC}}\langle\psi|\, \hat{\mathcal{O}}^{(\mathrm{A})} \,|\psi\rangle^{(\mathrm{A})}_{\mathrm{ABC}} = {}^{(\mathrm{A})}_{\mathrm{ABC}}\langle\psi|\, \left(\hat{\mathcal{S}}^{\mathrm{A}\to\mathrm{B}}\right)^{\dagger} \hat{\mathcal{S}}^{\mathrm{A}\to\mathrm{B}} \hat{\mathcal{O}}^{(\mathrm{A})} \left(\hat{\mathcal{S}}^{\mathrm{A}\to\mathrm{B}}\right)^{\dagger} \hat{\mathcal{S}}^{\mathrm{A}\to\mathrm{B}} \,|\psi\rangle^{(\mathrm{A})}_{\mathrm{ABC}} = {}^{(\mathrm{B})}_{\mathrm{ABC}}\langle\tilde{\psi}|\hat{\mathcal{O}}^{(\mathrm{B})}|\tilde{\psi}\rangle^{(\mathrm{B})}_{\mathrm{ABC}} \tag{6}$$

where $|\tilde{\psi}\rangle^{(\mathrm{B})}_{\mathrm{ABC}} = \hat{\mathcal{S}}^{\mathrm{A}\to\mathrm{B}}\,|\psi\rangle^{(\mathrm{A})}_{\mathrm{ABC}}$.

SUPPLEMENTARY NOTE 2: FAR-AWAY REFERENCE SYSTEM FOR WELL-DEFINED COORDINATE TIMES

In the main part, we assumed that the reference system R is far enough from the gravitational source that the gravitational pull of the mass on the reference frame can be neglected, at least for the duration of the experiment. As a consequence, we can omit the contribution of the reference system to the Hamiltonian. Moreover, this is necessary for defining a coordinate time t with respect to which the dynamical evolution of the remaining systems is described. As the point of this article is to at first be agnostic regarding the specific model for the gravitational field, it is important that we make this argument in a model-independent way. By "model independence", we mean that our argument is applicable to a vast range of models for the coupling of quantum matter to gravity and does not assume a specific one as a starting point. In particular, we should not assume that the reference system R "feels" a different semi-classical gravitational field in each branch. For instance, there are other models that suggest that a configuration in superposition sources an overall effective gravitational field. The goal of this supplementary note is thus to find bounds on the properties of the reference system R, independent of the specific model for the gravitational interaction.

First, the reference system R needs to be far enough from the mass configuration such that the change in relative distance due to the gravitational pull can be neglected over the time scale of the experiment. More concretely, let us consider the case of one point mass and R being initially at rest relative to the mass. We assume that, independently of the specific model for the gravitational interaction, the magnitude of the pull is *at most* as strong as the pull felt if the mass was only at the position closest to R. Denoting by d the relative distance to the closest mass position, we find that the relative distance of R changes at most by

$$\Delta r_{\mathrm{R}} = r_{\mathrm{R}}(\Delta t) - r_{\mathrm{R}}(0) = \left(d^{\frac{3}{2}} - 3\sqrt{\frac{MG}{2}}\Delta t\right)^{\frac{2}{3}} - d^{\frac{2}{3}}, \tag{7}$$

where we used the solution to the classical geodesic equation (7) in the Newtonian limit. Denoting the uncertainty in position of the reference system by Δx_R, we require that

$$|\Delta r_{\mathrm{R}}| < \Delta x_{\mathrm{R}} \tag{8}$$

so that the position of the reference system does not change *observably* during the duration Δt of the experiment. Note that the argument in this paper still requires well-localized reference systems in the sense that Δx_R cannot be too large. More specifically, for R to be a good reference system to describe the dynamics of the probe particle, it needs to be able to properly track its motion, that is

$$|\Delta r_{\mathrm{R}}| \ll |\Delta r_{\mathrm{S}}| \tag{9}$$

where Δr_{S} denotes the change in position of the probe particle during the time scale under consideration.

In models in which the reference system does not "experience" an effective gravitational field but a superposition thereof, we need to make further assumptions. First, the uncertainty in position of the reference system needs to be large enough to prevent it from getting entangled with the mass configuration. Thus, denoting by $\Delta r^{(i)}$ the change in position of the center of the wavepacket according to the gravitational field in the (i)-th branch, we require

$$|\Delta r^{(1)} - \Delta r^{(2)}| < \Delta x_{\mathrm{R}}. \tag{10}$$

Condition (10) is consistent with the observation made in [3] that the same time coordinates can be used to describe situations where the mass is placed in different positions.

Finally, we need to make sure that the reference system provides an operationally well-defined coordinate time t. This has been studied already in Ref. [4]. Modeling R as a clock as in "Application: Time Dilation", we require that

the internal states do not evolve significantly differently due to the gravitational field in each branch. In other words, their overlap should remain large over the time of the experiment:

$$|\langle s(\tau+\tau^{(1)})|s(\tau+\tau^{(2)})\rangle|^2 = \cos^2\left(\frac{E_0-E_1}{2}(\tau^{(1)}-\tau^{(2)})\right) \approx 1. \tag{11}$$

If this is satisfied, the time registered by R as a clock at the end of the experiment is practically the same in both branches. Thus, the internal state of R can be approximated as factorizing out and there is no observable entanglement with the position of the gravitational source.

SUPPLEMENTARY NOTE 3: EXPLICIT EXAMPLE OF A RELATIVE-DISTANCE-PRESERVING TRANSFORMATION

As an explicit example we consider four massive objects $\mathrm{M}_1,\ldots,\mathrm{M}_4$ in a superposition of two configurations with respect to a two-body system R. An additional particle S is placed in a definite position with respect to R. For illustration purposes, we restrict to two dimensions. As a consequence, the QRF change operator in Eq. (33) simplifies, requiring only two axes $\mathbf{a}$ and $\mathbf{b}$ instead of three to specify the reference frame of M. The two configurations in superposition are depicted in Supplementary Figure 1 below. Note that they are related by a rotation of an angle of 30° and no shift.

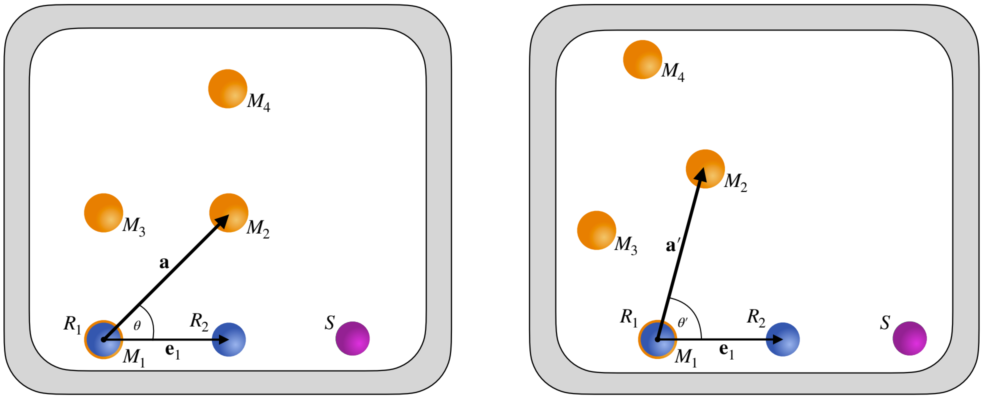

(a) Configuration in branch 1.   (b) Configuration in branch 2.

Supplementary Figure 1: Superposition of two mass configurations in the reference frame of R.

The quantum state with respect to R is

$$|\psi\rangle^{(\mathrm{R})}_{\mathrm{RMS}} = |\mathbf{0}\rangle_{\mathrm{R}_1}|\mathbf{e}_1\rangle_{\mathrm{R}_2}\otimes\frac{1}{\sqrt{2}}\Bigg(\left|\begin{pmatrix}0\\0\end{pmatrix}\right\rangle_{\mathrm{M}_1}\left|\begin{pmatrix}1\\1\end{pmatrix}\right\rangle_{\mathrm{M}_2}\left|\begin{pmatrix}0\\1\end{pmatrix}\right\rangle_{\mathrm{M}_3}\left|\begin{pmatrix}1\\2\end{pmatrix}\right\rangle_{\mathrm{M}_4} \tag{12}$$

$$+\left|\begin{pmatrix}0\\0\end{pmatrix}\right\rangle_{\mathrm{M}_1}\left|\frac{1}{2}\begin{pmatrix}\sqrt{3}-1\\\sqrt{3}+1\end{pmatrix}\right\rangle_{\mathrm{M}_2}\left|\frac{1}{2}\begin{pmatrix}-1\\\sqrt{3}\end{pmatrix}\right\rangle_{\mathrm{M}_3}\left|\frac{1}{2}\begin{pmatrix}-2+\sqrt{3}\\1+2\sqrt{3}\end{pmatrix}\right\rangle_{\mathrm{M}_4}\Bigg)\otimes\left|\begin{pmatrix}2\\0\end{pmatrix}\right\rangle_{\mathrm{S}}, \tag{13}$$

where $\mathbf{e}_1 = (1,0)^T$ is the unit vector in $\mathbf{x}_1$-direction. In order to change into the reference frame of M, we need to apply the operator $\hat{\mathcal{S}}^{\mathrm{R}\to\mathrm{M}}$ as defined in Eq. (33). This consists of several steps. First, we have to change to relative coordinates. Since M_1 is already at the origin, the axes defined by system M are simply $\mathbf{a}=\mathbf{x}_2$ and $\mathbf{b}=\mathbf{x}_3$ and similarly for the second branch. Writing the position vectors of M_4 as a linear combination of these two vectors, we find the relative degrees of freedom $r_4^1 = r_4^2 = 1$ in both branches. Consequently, they factorize out. Secondly, we apply the operator $\hat{U}_{\mathrm{MSR}}$, which adjusts R to match the length of the direction-vectors defined by system M: $\mathbf{e}_1 \to \mathbf{f}_1 \equiv (\sqrt{2},0)^T$. Furthermore, $\mathbf{b}$ and the position vector of particle S are rotated by the angle $\theta(\mathbf{e}_1,\mathbf{a}) = 45°$ in the first and by $\theta(\mathbf{e}_1,\mathbf{a}') = 75°$ in the second branch. Applying $\hat{\mathcal{P}}_{\mathrm{MR}}$ and transforming back to the original coordinates,

we obtain the state

$$|\psi\rangle^{(\mathrm{M})}_{\mathrm{MRS}} = \hat{\mathcal{S}}^{\mathrm{R}\to\mathrm{M}} \, |\psi\rangle^{(\mathrm{R})}_{\mathrm{RMS}} = |\mathbf{0}\rangle_{\mathrm{M}_1} \, |\mathbf{f}_1\rangle_{\mathrm{M}_2} \left| \frac{1}{\sqrt{2}} \begin{pmatrix} 1 \\ 1 \end{pmatrix} \right\rangle_{\mathrm{M}_3} \left| \frac{1}{\sqrt{2}} \begin{pmatrix} 3 \\ 1 \end{pmatrix} \right\rangle_{\mathrm{M}_4} \otimes \tag{14}$$

$$|\mathbf{0}\rangle_{\mathrm{R}_1} \otimes \frac{1}{\sqrt{2}} \left(\left| \begin{pmatrix} 1 \\ -1 \end{pmatrix} \right\rangle_{\mathrm{R}_2} \left| \sqrt{2} \begin{pmatrix} 1 \\ -1 \end{pmatrix} \right\rangle_{\mathrm{S}} + \left| \frac{1}{2} \begin{pmatrix} \sqrt{3}-1 \\ -\sqrt{3}-1 \end{pmatrix} \right\rangle_{\mathrm{R}_2} \left| \frac{1}{\sqrt{2}} \begin{pmatrix} \sqrt{3}-1 \\ -\sqrt{3}-1 \end{pmatrix} \right\rangle_{\mathrm{S}} \right) \tag{15}$$

with respect to the reference frame of system M. The resulting configurations are depicted in Supplementary Figure 2 below.

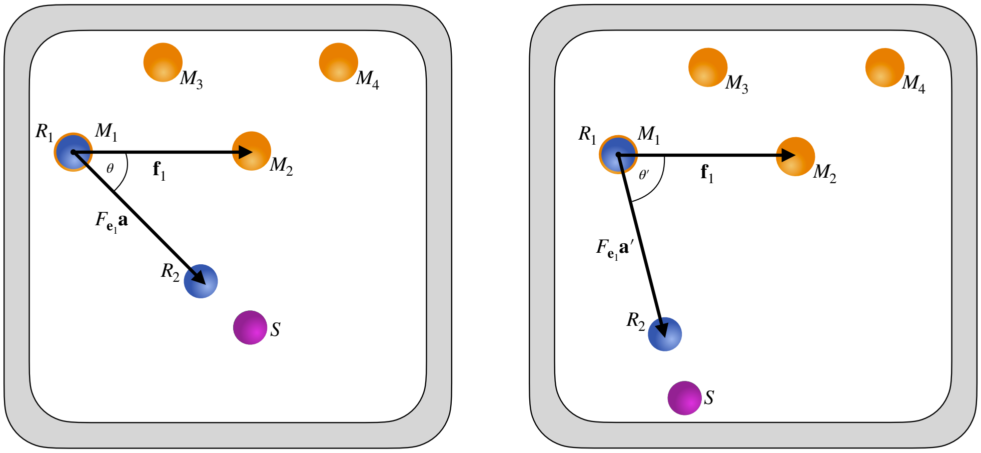

(a) Configuration in branch 1. (b) Configuration in branch 2.

Supplementary Figure 2: Superposition of two mass configurations in the reference frame of M.

SUPPLEMENTARY NOTE 4: COMPARISON WITH PERTURBATIVE APPROACHES TO QUANTUM GRAVITY

In this Supplementary Note, we discuss to what extent the approach presented in this work goes beyond the perturbative approaches to quantum gravity. To this end, let us begin by reviewing the perturbative approach, following Chapters 1 and 2 of Ref. [5]. Generally, it is possible to expand the metric tensor $g_{\mu\nu}$ around a fixed metric. Often, this is done around the flat Minkowski metric and with the gravitational coupling $\kappa = \sqrt{32\pi G}$ (for $\hbar = c = 1$) as an expansion parameter, such that

$$g_{\mu\nu} = \eta_{\mu\nu} + \kappa h_{\mu\nu}, \tag{16}$$

where $h_{\mu\nu}$ is referred to as the metric perturbation. An expansion in the perturbation $\kappa h_{\mu\nu}$ and a neglect of higher order terms is only valid if the latter is "small" compared to the background metric. As a concrete example, take the Schwarzschild metric sourced by an object with mass M,

$$g = -\left(1 - \frac{2GM}{r}\right) dt^2 + \left(1 - \frac{2GM}{r}\right)^{-1} dr^2 + r^2 g_\Omega, \tag{17}$$

where g_Ω denotes the metric on the two-sphere. Let us now see at which scales perturbation theory around the flat Minkowski metric is valid and when it breaks down. The time-time component of the Schwarzschild metric $g^{00} = (1 - \frac{2GM}{r})$ can be seen as a perturbation around the time-time component of the Minkowski metric $g^{00} = 1$ if $\frac{M}{r} \ll \frac{1}{2G} = \frac{1}{2}M_{\mathrm{Pl}}^2$ where M_{Pl} denotes the Planck mass. Thus, when the ratio of the mass M of and the distance r from the gravitational source approaches $\frac{1}{2}M_{\mathrm{Pl}}^2$, the perturbative contribution grows too large and perturbation theory loses its validity. Importantly, this means that perturbation theory breaks down exactly at the Schwarzschild radius $r_{\mathrm{s}} = 2GM$ as this is the distance at which $M/r_{\mathrm{s}} = 1/(2G)$ for any massive object. However, already outside of the Schwarzschild radius but in its vicinity, perturbation theory would no longer provide a good approximation to the full theory as the higher order terms contribute significantly to the expansion.

More generally, in a perturbative approach to quantum gravity, one can view $h_{\mu\nu}$ as a quantum field on flat spacetime. In this case, an expansion in the coupling constant κ will be valid only at certain energy scales. In particular, κ has dimension $\sqrt{G} = 1/M_{\text{Pl}}$. Thus, this perturbative approach to quantum gravity is valid only for energy scales smaller than $M_{\text{Pl}}c^2 \approx 10^{18}\text{GeV}$. In other words, one can neglect terms of higher order in κ only as long as the energy scale is below the Planck mass.

In contrast, the approach devised in our work does not rely on perturbative methods at any point and is thus limited to the realms discussed above. By restricting the set of configurations that we treat to superpositions of mass configurations related by global translations and rotations, we are able to make predictions, solely based on the assumption of covariance under quantum reference frame transformations. This assumption allows us to map a situation in which we have a gravitational source in a superposition of two or more locations to one in which it is in a well-localized position. We thus avoid any mathematical treatment of the "quantum metric" sourced by such a configuration. On the contrary, we can use classical general relativity to determine the spacetime geometry and consequently the motion of test particles and clocks. Therefore, we only rely on the regime of validity of full general relativity. In particular, our model makes predictions for realms in which perturbation theory breaks down. That is, if the metrics sourced by the gravitating object in each of the branches separately cannot be obtained by perturbations around the *same* background, our approach can make predictions whereas perturbation theory cannot be applied. Concretely, consider a probe particle in the presence of a black hole in a superposition of two distant locations – one close to and one far away from the probe. If we wanted to apply linearized quantum gravity, we would have to describe the gravitational field at the location of the probe particle as a superposition of perturbations around the *same* spacetime background g^0. However, if the superposition of the black hole is large enough, there does not exist a g^0 such that

$$g^1(x_{\text{p}}) = g^0_{\mu\nu}(x_{\text{p}}) + h^1_{\mu\nu}(x_{\text{p}}), \tag{18}$$

$$g^2(x_{\text{p}}) = g^0_{\mu\nu}(x_{\text{p}}) + h^2_{\mu\nu}(x_{\text{p}}), \tag{19}$$

where $h^1, h^2 \ll g^0$. Our approach, on the other hand, allows to change to a quantum frame in which the black hole is localized at one definite position, thus sourcing a classical gravitational field in which the dynamics of the probe particle can be computed. Note, however, that while the present approach goes beyond the regime of applicability of perturbative approaches as explained above, it does not yet take into account any quantum fluctuations of spacetime. In this regard, our approach is *complementary* to perturbative approaches to quantum gravity, which include quantum fluctuations of the perturbation.

Supplementary References

[1] A.-C. de la Hamette and T. D. Galley, Quantum reference frames for general symmetry groups, Quantum **4**, 367 (2020).

[2] F. Giacomini, E. Castro-Ruiz, and Č. Brukner, Quantum mechanics and the covariance of physical laws in quantum reference frames, Nature Communications **10**, 494 (2019).

[3] M. Zych, F. Costa, I. Pikovski, and Č. Brukner, Bell's theorem for temporal order, Nature Communications **10**, 3772 (2019).

[4] E. Castro Ruiz, F. Giacomini, and Č. Brukner, Entanglement of quantum clocks through gravity, Proceedings of the National Academy of Sciences **114**, E2303–E2309 (2017).

[5] M. Maggiore, *Gravitational Waves. Vol. 1: Theory and Experiments*, Oxford Master Series in Physics (Oxford University Press, 2007).
